# Supplementary material for: Mucochytrium quahogii (=QPX) Is a Commensal, Opportunistic Pathogen of the Hard Clam (Mercenaria mercenaria): Evidence and Implications for QPX Disease Management
Source: J Fungi (Basel). 2022 Oct 26;8(11):1128. doi: 10.3390/jof8111128 (PMC9695251; doi:10.3390/jof8111128)
Supplement: Supplementary file 1 [file jof-08-01128-s001.zip › jof-1917028-supplementary.pdf]

# Supplementary Figures and Tables

*Journal of Fungi (ISSN 2309-608X) – special issue "Fungal infections in fishes and aquatic invertebrates/fungal pathogenesis and disease control"*

*Research Article*

## *Mucochytrium quahogii* (=QPX) is a commensal, opportunistic pathogen of the hard clam (*Mercenaria mercenaria*): evidence and implications for QPX disease management

Sabrina Geraci-Yee, Jackie L. Collier and Bassem Allam*

| | |
|---|---|
| Supplemental Figures | p. 2–6 |
| Figure S1 | p. 2 |
| Figure S2 | p. 3 |
| Figure S3 | p. 4 |
| Figure S4 | p. 5 |
| Figure S5 | p. 6 |
| Figure S6 | p. 6 |
| | |
| Supplemental Tables | p. 7–18 |
| Table S1 | p. 7–8 |
| Table S2 | p. 9 |
| Table S3 | p. 9 |
| Table S4 | p. 10 |
| Table S5 | p. 10 |
| Table S6 | p. 11 |
| Table S7 | p. 12 |
| Table S8 | p. 13 |
| Table S9 | p. 14 |
| Table S10 | p. 15 |
| Table S11 | p. 16–17 |
| Table S12 | p. 18 |

## Supplementary Figures

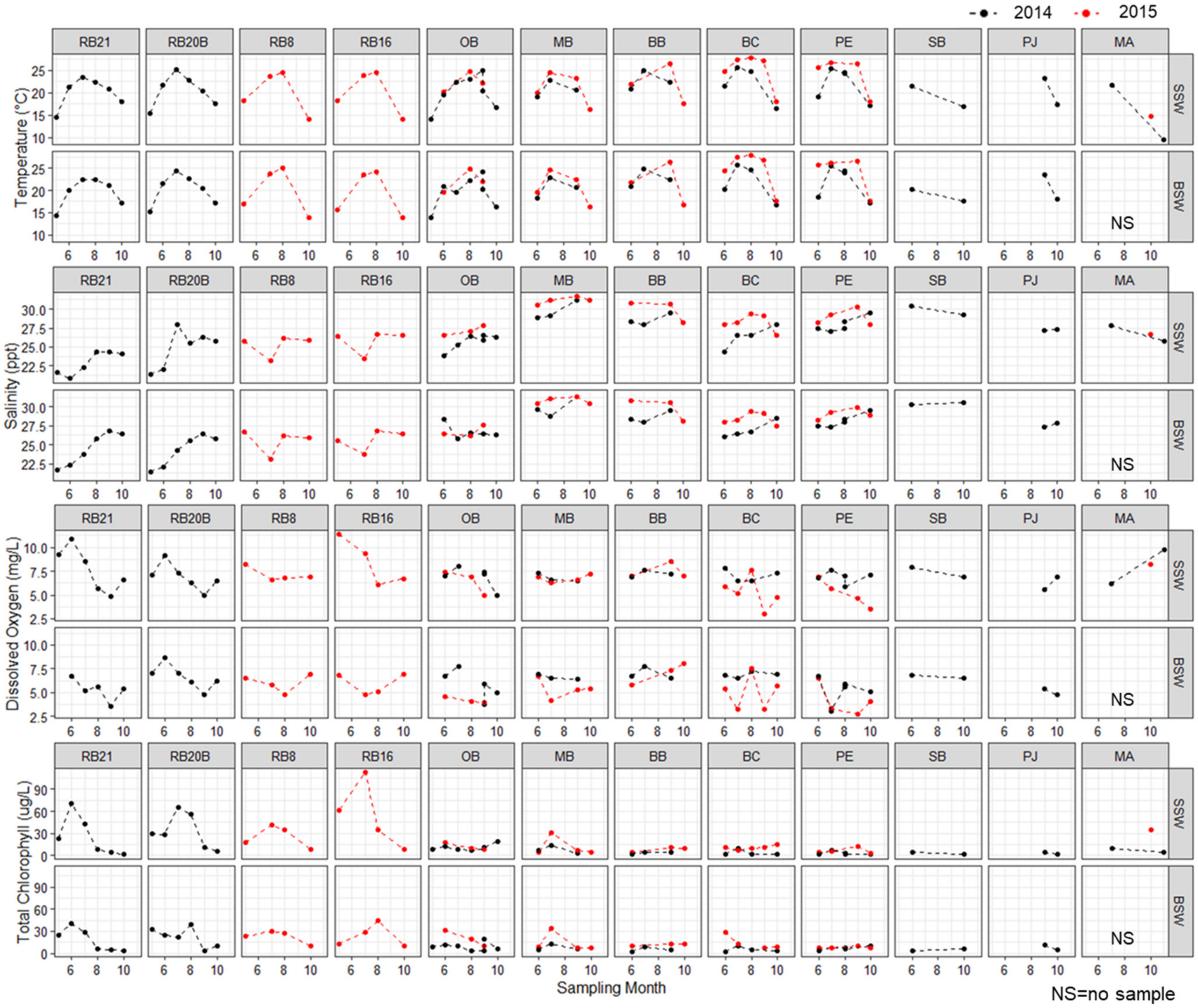


**Figure S1:** Measured environmental parameters by site and year for surface (SSW) and bottom (BSW) seawater.

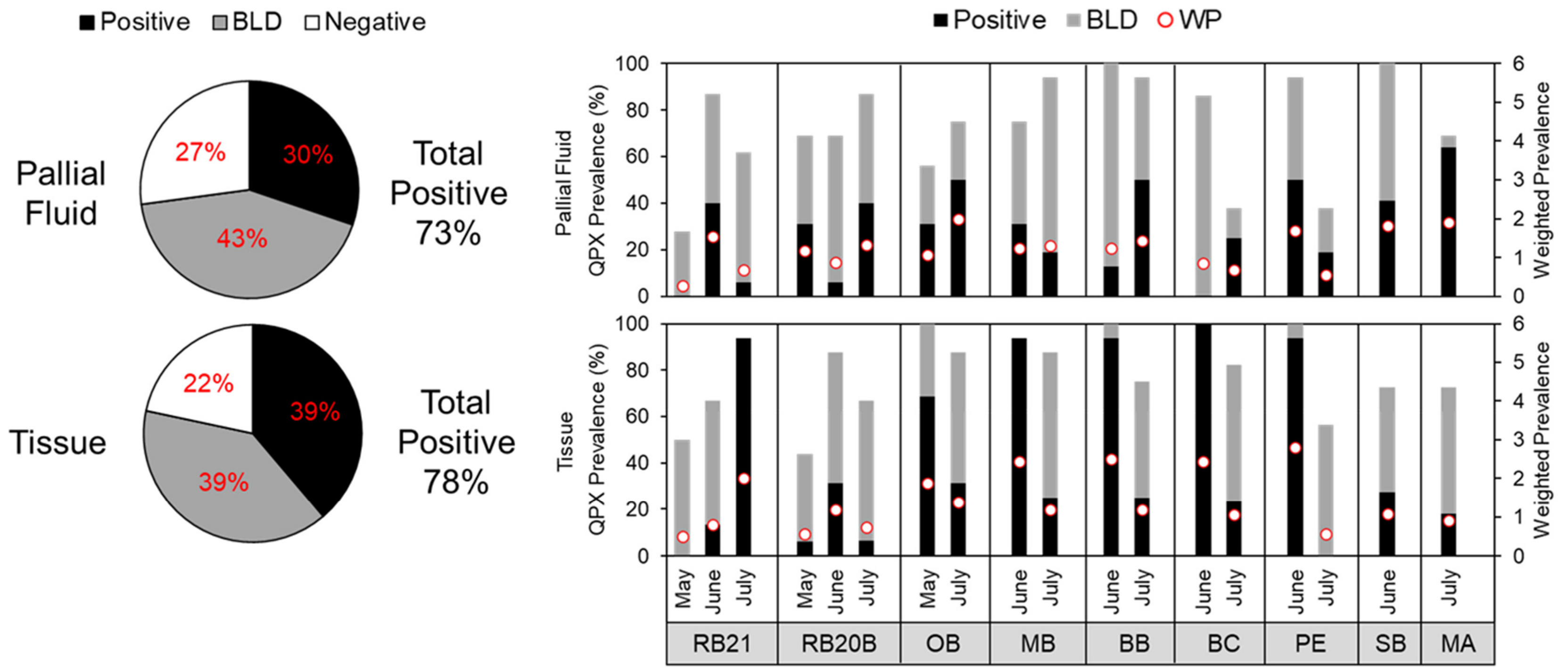


**Figure S2:** Summary of QPX prevalence in hard clam pallial fluid and mantle tissue field samples. Total QPX prevalence is the sum of positive and BLD samples.

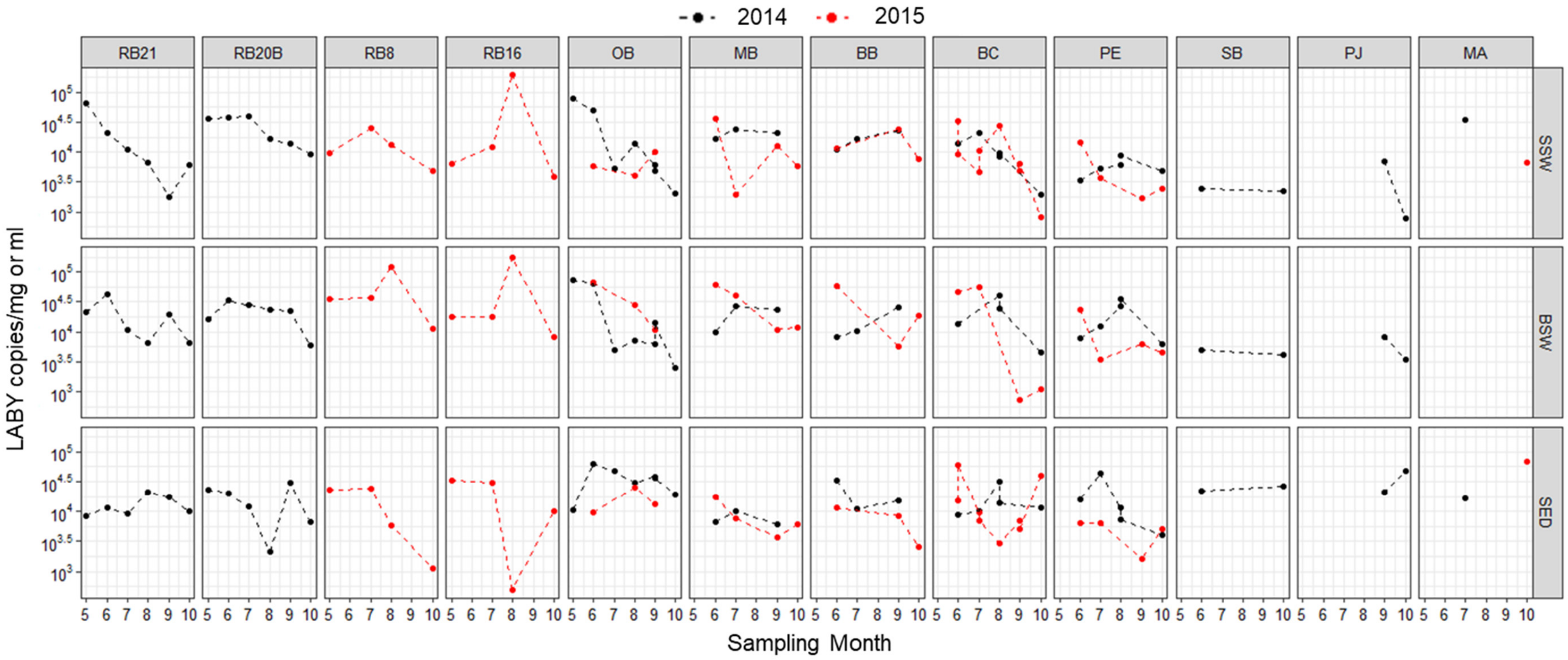


**Figure S3:** Labyrinthulomycete (LABY) abundance in environmental samples: surface seawater (SSW), bottom seawater (BSW), and sediment (SED), assayed using the labyrinthulomycete (LABY) qPCR. Values are expressed in terms of LABY gene copies per mL seawater or mg sediment on a log10 scale.

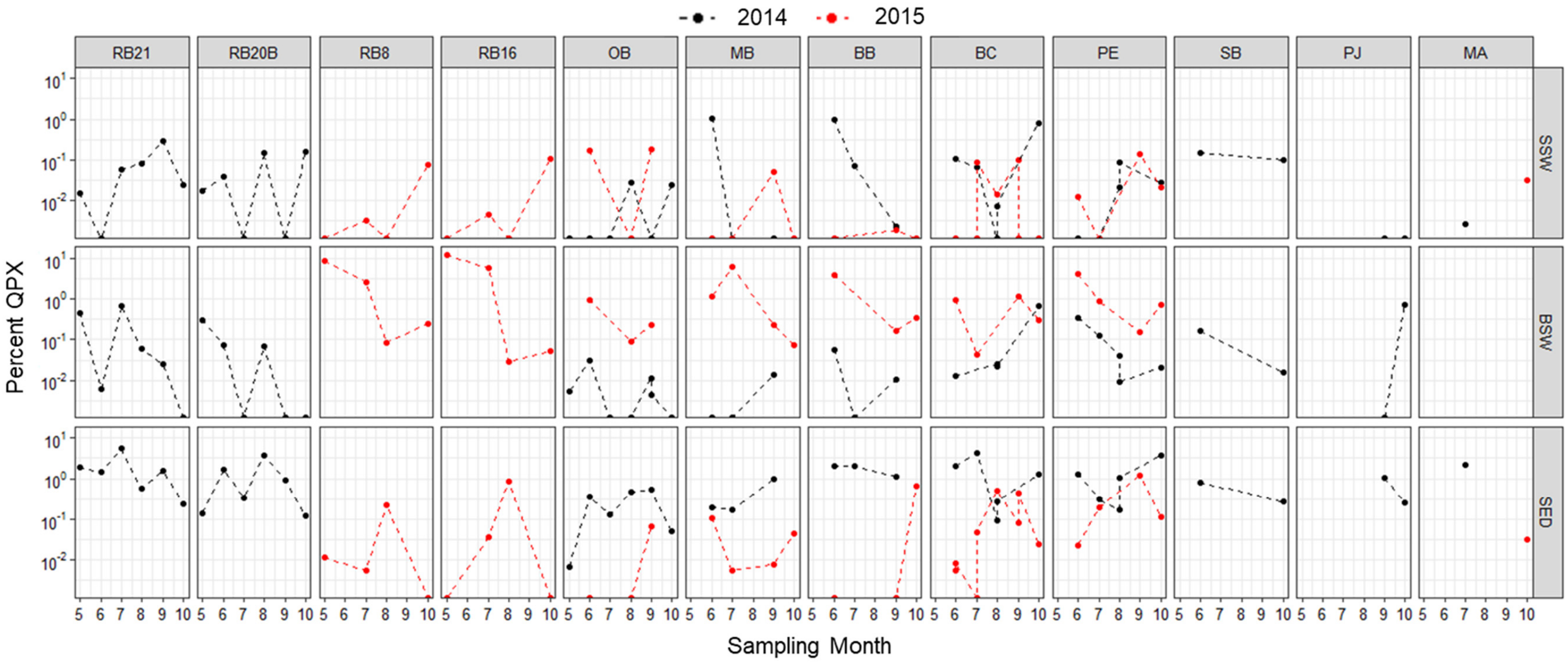


**Figure S4:** Percent contribution of *M. quahogii* (QPX) to total labyrinthulomycetes in environmental samples: surface seawater (SSW), bottom seawater (BSW), and sediment (SED) on a log10 scale.

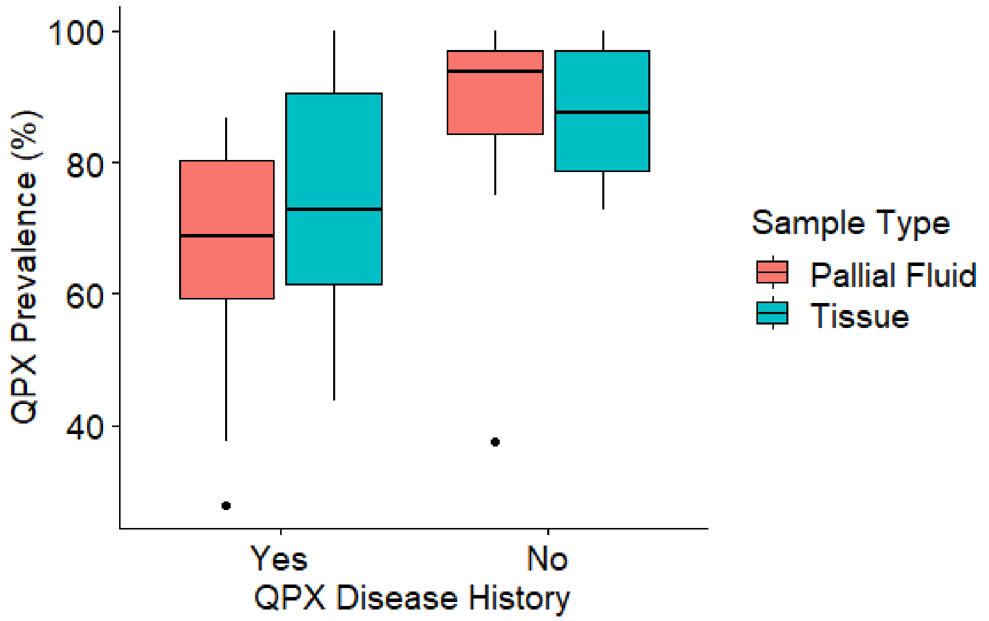


**Figure S5:** *M. quahogii* (QPX) prevalence (%TPOS = total positive included positive and BLD samples determined by qPCR) in hard clam pallial fluid and tissue ($n$ = 291 subset only for tissue) grouped by sites with and without a history of QPX disease. The difference between pallial fluid samples was significant ($p$ = 0.0229) without $p$-value adjustment by Wilcoxon rank sum test, while the difference between tissue samples was not ($p$ = 0.201).

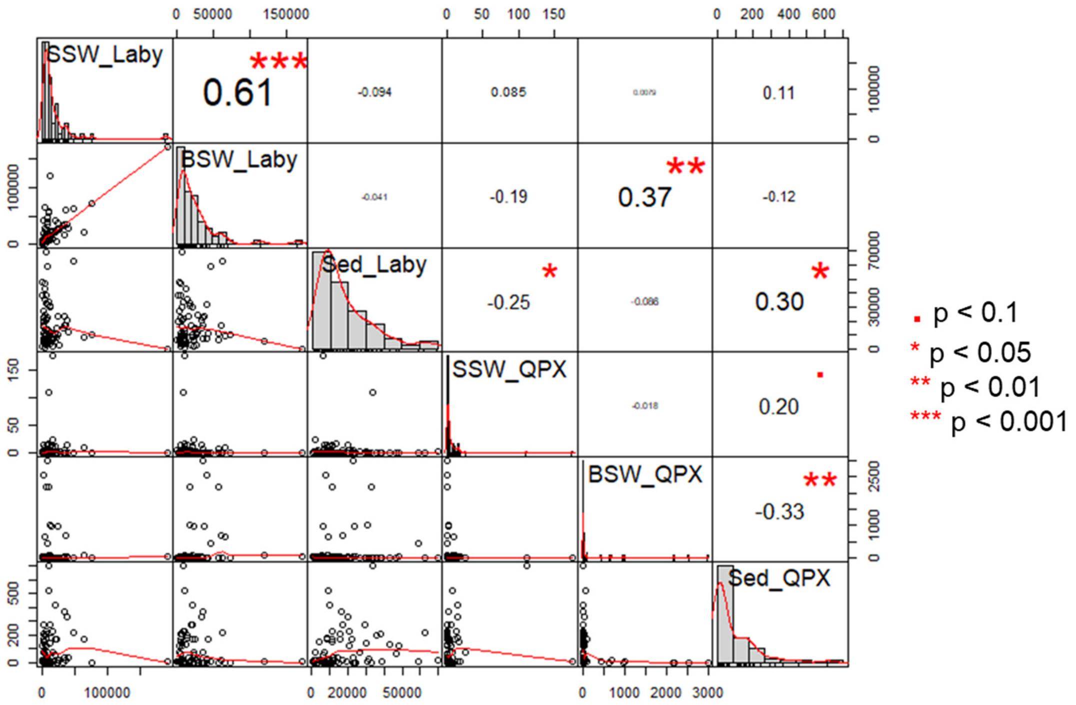


**Figure S6:** Spearman correlation coefficients (rho) correlogram with histograms for QPX and labyrinthulomycetes in the environment.

## Supplementary Tables

**Table S1:** Sampling sites and samples collected during the field survey in 2014 and 2015.

| Sample | Date | Site | Sample Type |
|---|---|---|---|
| 1 | 5/13/2014 | Oyster Bay | Clams + Environmental |
| 2 | 5/20/2014 | Raritan Bay 21 | Clams + Environmental |
| 3 | 5/20/2014 | Raritan Bay 20B | Clams + Environmental |
| 4 | 6/3/2014 | Birch Creek | Clams + Environmental |
| 5 | 6/3/2014 | Peconic Estuary | Clams + Environmental |
| 6 | 6/5/2014 | Babylon | Clams + Environmental |
| 7 | 6/5/2014 | Moriches Bay | Clams + Environmental |
| 8 | 6/10/2014 | Oyster Bay | Environmental |
| 9 | 6/17/2014 | Raritan Bay 21 | Clams + Environmental |
| 10 | 6/17/2014 | Raritan Bay 20B | Clams + Environmental |
| 11 | 6/24/2014 | Shinnecock Bay | Clams + Environmental |
| 12 | 7/1/2014 | Birch Creek | Clams + Environmental |
| 13 | 7/1/2014 | Peconic Estuary | Clams + Environmental |
| 14 | 7/7/2014 | Oyster Bay | Clams + Environmental |
| 15 | 7/15/2014 | MA | Clams + Environmental |
| 16 | 7/17/2014 | Moriches Bay | Clams + Environmental |
| 17 | 7/17/2014 | Babylon | Clams + Environmental |
| 18 | 7/22/2014 | Raritan Bay 21 | Clams + Environmental |
| 19 | 7/22/2014 | Raritan Bay 20B | Clams + Environmental |
| 20 | 8/4/2014 | Oyster Bay | Environmental |
| 21 | 8/5/2014 | Peconic Estuary | Environmental |
| 22 | 8/5/2014 | Birch Creek | Environmental |
| 23 | 8/19/2014 | Raritan Bay 21 | Clams + Environmental |
| 24 | 8/19/2014 | Raritan Bay 20B | Clams + Environmental |
| 25 | 8/26/2014 | Peconic Estuary | Clams + Environmental |
| 26 | 8/26/2014 | Birch Creek | Clams + Environmental |
| 27 | 9/2/2014 | Oyster Bay | Clams + Environmental |
| 28 | 9/9/2014 | Port Jefferson Harbor | Clams + Environmental |
| 29 | 9/12/2014 | Babylon | Environmental |
| 30 | 9/12/2014 | Moriches Bay | Environmental |
| 31 | 9/16/2014 | Raritan Bay 21 | Clams + Environmental |
| 32 | 9/16/2014 | Raritan Bay 20B | Clams + Environmental |
| 33 | 9/30/2014 | Oyster Bay | Clams + Environmental |
| 34 | 10/6/2014 | Peconic Estuary | Environmental |
| 35 | 10/6/2014 | Birch Creek | Environmental |
| 36 | 10/6/2014 | Shinnecock Bay | Clams + Environmental |
| 37 | 10/15/2014 | Raritan Bay 21 | Clams + Environmental |
| 38 | 10/15/2014 | Raritan Bay 20B | Clams + Environmental |
| 39 | 10/21/2014 | Oyster Bay | Clams + Environmental |
| 40 | 10/21/2014 | Port Jefferson Harbor | Environmental |
| 41 | 11/14/2014 | MA | Clams |
| 42 | 5/27/2015 | Raritan Bay 8 | Clams + Environmental |

**Table S1 (cont'd):** Sampling sites and samples collected during the field survey in 2014 and 2015.

| Sample | Date | Site | Sample Type |
|---|---|---|---|
| 43 | 5/27/2015 | Raritan Bay 16 | Clams + Environmental |
| 44 | 6/4/2015 | Birch Creek | Environmental |
| 45 | 6/11/2015 | Babylon Bay | Clams + Environmental |
| 46 | 6/11/2015 | Moriches Bay | Clams + Environmental |
| 47 | 6/17/2015 | Oyster Bay | Clams + Environmental |
| 48 | 6/23/2015 | Birch Creek | Clams + Environmental |
| 49 | 6/23/2015 | Peconic Bay | Clams + Environmental |
| 50 | 7/7/2015 | Raritan Bay 8 | Clams + Environmental |
| 51 | 7/7/2015 | Raritan Bay 16 | Clams + Environmental |
| 52 | 7/14/2015 | Birch Creek | Environmental |
| 53 | 7/22/2015 | Moriches Bay | Clams + Environmental |
| 54 | 7/28/2015 | Peconic Estuary | Clams + Environmental |
| 55 | 7/28/2015 | Birch Creek | Clams + Environmental |
| 56 | 8/10/2015 | Oyster Bay | Clams + Environmental |
| 57 | 8/18/2015 | Birch Creek | Environmental |
| 58 | 8/26/2015 | Raritan Bay 8 | Clams + Environmental |
| 59 | 8/26/2015 | Raritan Bay 16 | Clams + Environmental |
| 60 | 9/2/2015 | Babylon Bay | Clams + Environmental |
| 61 | 9/2/2015 | Moriches Bay | Clams + Environmental |
| 62 | 9/9/2015 | Peconic Estuary | Clams + Environmental |
| 63 | 9/9/2015 | Birch Creek | Clams + Environmental |
| 64 | 9/22/2015 | Oyster Bay | Clams + Environmental |
| 65 | 9/29/2015 | Birch Creek | Environmental |
| 66 | 10/7/2015 | Babylon Bay | Clams + Environmental |
| 67 | 10/7/2015 | Moriches Bay | Clams + Environmental |
| 68 | 10/13/2015 | Peconic Estuary | Clams + Environmental |
| 69 | 10/13/2015 | Birch Creek | Clams + Environmental |
| 70 | 10/16/2015 | MA | Clams + Environmental |
| 71 | 10/20/2015 | Raritan Bay 8 | Clams + Environmental |
| 72 | 10/20/2015 | Raritan Bay 16 | Clams + Environmental |

**Table S2:** Intensity scale used to calculate *M. quahogii* (QPX) weighted prevalence in hard clam cohort mantle tissue and pallial fluid based on qPCR assays. Weighted prevalence was determined based on the sum of QPX load rated on the intensity scale for each individual clam, divided by the number of clams assayed for each sampling event or cohort. Scales differ due to different detection limit of the two assays.

| **Intensity** | **Tissue QPX copies/mg** | **Pallial Fluid QPX copies/ml** |
|---|---|---|
| 0 = None | 0 = negative | 0 = negative |
| 1 = Rare | < 75 = BLD | < 500 = BLD |
| 2 = Light | 440 | 880 |
| 3 = Mild | 2200 | 2200 |
| 4 = Moderate | 11,000 | 11,000 |
| 5 = Heavy | 55,000 | 55,000 |
| 6 = Severe | > 55,000 | > 55,000 |

BLD = below limit of detection

**Table S3:** Local weather stations used to supplement measured metadata.

| **Sampling Site** | **Coordinates** | **Weather Station** |
|---|---|---|
| Raritan Bay 21 | 40.498917, −74.1797 | Newark, NJ (KEWR) |
| Raritan Bay 20B | 40.542917, −74.1289 | Newark, NJ (KEWR) |
| Raritan Bay 8 | 40.501867, −74.1869 | Newark, NJ (KEWR) |
| Raritan Bay 16 | 40.50625, −74.1526 | Newark, NJ (KEWR) |
| Oyster Bay | 40.8993, −73.4897 | Farmingdale, NY (KFRG) |
| Moriches Bay | 40.777283, −72.7912 | Shirley, NY (KHWV) |
| Babylon Bay | 40.665983, −73.3123 | Farmingdale, NY (KFRG) |
| Birch Creek | 40.9044, −72.5904 | East Quogue, NY (KFOK) |
| Peconic Estuary | 40.941617, −72.4137 | East Quogue, NY (KFOK) |
| Shinnecock Bay | 40.86195, −72.4483 | East Quogue, NY (KFOK) |
| Port Jefferson Harbor | 40.953117, −73.0702 | Ronkonkoma, NY (KISP) |
| Barnstable, MA | 41.709798, −70.320348 | Hyannis, MA (KHYA) |

**Table S4:** Descriptive statistics of *M. quahogii* (QPX) prevalence (%) and concentration (copies/mg) in hard clam tissue at the cohort level (not individual clams, *n* = 59) determined by qPCR.

| Hard Clam Tissue | | Mean | Standard Error | Median | Standard Deviation | Range | Minimum | Minimum (non-zero) | Maximum | Confidence Interval (95.0%) |
|---|---|---|---|---|---|---|---|---|---|---|
| Prevalence | TPOS | 74.7 | 3 | 81.25 | 23.07 | 85 | 15 | 15 | 100 | 6.01 |
| | POS | 30.9 | 4.03 | 18.75 | 30.97 | 100 | 0 | 5 | 100 | 8.07 |
| | BLD | 43.8 | 2.73 | 50 | 20.96 | 87.5 | 0 | 6.25 | 87.5 | 5.46 |
| | NEG | 25.3 | 3 | 18.75 | 23.06 | 85 | 0 | 6.25 | 85 | 6.01 |
| | WP | 1.15 | 0.08 | 1 | 0.64 | 2.66 | 0.15 | 0.15 | 2.81 | 0.17 |
| Concentration | MEAN | 1621 | 1045 | 150.29 | 8026 | 60,342 | 0 | 82.26 | 60,342 | 2092 |
| | MIN | 125.64 | 27.03 | 81.81 | 207.62 | 1254 | 0 | 75.3 | 1254 | 54.11 |
| | MAX | 13,616 | 11,249 | 246.34 | 86,406 | 66,0824 | 0 | 84.75 | 660,824 | 22,518 |
| | RANGE | 13,491 | 11,250 | 126.56 | 86,412 | 660,739 | 0 | 3.2 | 660,739 | 22,519 |

TPOS = % total positive (includes POS + BLD)
POS = % positive (quantifiable)
BLD = % below limit of detection (positive but unquantifiable)
NEG = % negative
WP = weighted prevalence
MIN = minimum concentration of QPX (copies/mg tissue)
MAX = maximum concentration of QPX (copies/mg tissue)
MEAN = mean concentration of QPX (copies/mg tissue) of positive samples only (excludes BLD and NEG)
RANGE = concentration range of QPX (copies/mg tissue)

**Table S5:** Hard clam samples positive for QPX disease by histopathology.

| Sample | Site | Month | QPX copies/mg | QPX cells/mg | Intensity | Location |
|---|---|---|---|---|---|---|
| 14-041-15 | RB21 | September | 234.60 | 0.53 | Moderate, multifocal | Visceral Mass and Gills |
| 15-011-13 | RB8 | May | 8845.29 | 20.10 | Moderate, multifocal | Gills and Mantle |
| 15-012-13 | RB16 | May | 5366.30 | 12.20 | Light, multifocal | Visceral Mass |
| 15-046-01 | RB8 | August | 1664.75 | 3.78 | Rare, focal | Visceral Mass |
| 15-061-28 | RB16 | October | 1092.90 | 2.48 | Light, multifocal | Mantle |
| 15-059-08 | MA | October | 60338.27 | 137.13 | Light, focal | Gills |
| 15-059-13 | MA | October | 83826.37 | 190.51 | Moderate, multifocal | Mantle |

**Table S6:** Descriptive statistics of *M. quahogii* (QPX) prevalence (%) and concentration (copies/mL) in hard clam pallial fluid at the cohort level (not individual clams, n=18) determined by qPCR.

| Hard Clam Pallial Fluid | | Mean | Standard Error | Median | Standard Deviation | Range | Minimum | Minimum (non-zero) | Maximum | Confidence Interval (95.0%) |
|---|---|---|---|---|---|---|---|---|---|---|
| Prevalence | TPOS | 73.19 | 5.21 | 75 | 22.11 | 72.22 | 27.78 | 27.78 | 100 | 10.99 |
| | POS | 28.66 | 4.48 | 31.25 | 19 | 63.64 | 0 | 6.25 | 63.64 | 9.45 |
| | BLD | 44.54 | 5.58 | 43.75 | 23.68 | 82.95 | 4.55 | 4.55 | 87.50 | 11.78 |
| | NEG | 26.81 | 5.21 | 25 | 22.11 | 72.22 | 0 | 6.25 | 72.22 | 10.99 |
| | WP | 1.21 | 0.11 | 1.25 | 0.48 | 1.72 | 0.28 | 0.28 | 2 | 0.24 |
| Concentration | MEAN | 1587 | 445 | 1199 | 1889 | 8655 | 0 | 515 | 8655 | 939 |
| | MIN | 615 | 113 | 552 | 480 | 2351 | 0 | 502 | 2351 | 239 |
| | MAX | 5121 | 2850 | 2442 | 12,092 | 53,080 | 0 | 515 | 53,080 | 6013 |
| | RANGE | 4506 | 2852 | 1727 | 12,102 | 52,537 | 0 | 410 | 52,537 | 6018 |

TPOS = % total positive (includes POS + BLD)
POS = % positive (quantifiable)
BLD = % below limit of detection (positive but unquantifiable)
NEG = % negative
WP = weighted prevalence
MIN = minimum concentration of QPX (copies/ml pallial fluid)
MAX = maximum concentration of QPX (copies/ml pallial fluid)
MEAN = mean concentration of QPX (copies/ml pallial fluid) of positive samples only (excludes BLD and NEG)
RANGE = concentration range of QPX (copies/ml pallial fluid)

**Table S7:** Descriptive statistics of *M. quahogii* (QPX) in environmental samples, determined by qPCR in QPX gene copies/mg sediment or ml seawater. SED = sediment; BSW = bottom seawater; SSW = surface seawater.

| Statistic | SED | BSW | SSW |
|---|---|---|---|
| Count (N) | 71 | 64 | 71 |
| % Positive | 89 | 83 | 56 |
| Mean | 93.15 | 241.44 | 7.36 |
| Standard Error | 16.06 | 78.81 | 2.92 |
| Median | 22.1 | 9.84 | 0.54 |
| Standard Deviation | 135.3 | 630.5 | 24.64 |
| Range | 702.61 | 2980 | 176.37 |
| Minimum | 0 | 0 | 0 |
| Minimum (non-zero) | 0.27 | 0.61 | 0.43 |
| Maximum | 702.61 | 2980 | 176.37 |
| Confidence Interval (95.0%) | 32.03 | 157.49 | 5.83 |

**Table S8:** *p*-values of group comparisons of *M. quahogii* in clams and environment by Wilcoxon rank sum test or Kruskal-Wallis rank sum test. *p*-values in bold were significant after Bonferroni (BF) correction* for each set of comparisons. *p*-values in red were significant without BF correction for exploratory analyses.

| Sample | Parameter | Wilcoxon Rank Sum Test | | Kruskal-Wallis Rank Sum Test | |
|---|---|---|---|---|---|
| | | QPX Disease History | Year | Site | Month |
| Clam Tissue (sig at $p < 0.0125$) | TPOS | 0.2456 | 0.1139 | 0.2186 | 0.1340 |
| | POS | 0.1693 | 0.1861 | 0.2257 | 0.0356 |
| | BLD | 0.9937 | 0.0662 | 0.8251 | 0.1519 |
| | WP | 0.0963 | 0.1824 | 0.2286 | 0.0411 |
| Clam Pallial Fluid (sig at $p < 0.0125$) | TPOS | 0.0229 | n/a | 0.362 | 0.0446 |
| | POS | 0.439 | | 0.467 | 0.549 |
| | BLD | 0.364 | | 0.433 | 0.0444 |
| | WP | 0.341 | | 0.473 | 0.322 |
| Environment (sig at $p < 0.0167$) | SED | 0.8438 | **$6.25 \times 10^{-11}$** | 0.5372 | 0.5563 |
| | BSW | 0.8694 | **$6.24 \times 10^{-5}$** | 0.6601 | 0.0636 |
| | SSW | 0.4396 | 0.0866 | 0.7298 | 0.9564 |

*$p$ = 0.05/ 4 comparisons for clams = 0.0125; $p$ = 0.05/3 comparisons for environmental (ENV) = 0.0167; TPOS includes positive and BLD samples

**Table S9:** Significant *p*-values of metadata by Wilcoxon rank sum test grouped by sampling year without adjustment for multiple comparisons. *p*-values in bold were significant after Bonferroni* (BF) correction.

| **Metadata** | ***p*-value** |
|---|---|
| SSW Salinity | 0.01326 |
| Δ Salinity (SSW – BSW) | **0.0001487** |
| SSW Total chlorophyll | 0.03183 |
| BSW Total chlorophyll | 0.01831 |
| BSW Chlorophyll a | 0.009892 |
| Wind Degree Direction | 0.0401 |
| Precipitation (mean 3 month) | **0.001011** |
| Precipitation (mean 4 month) | **0.0001211** |
| Precipitation (sum 2 month) | 0.04803 |
| Precipitation (sum 3 month) | **0.0009504** |
| Precipitation (sum 4 month) | **$50.6 \times 10^{-5}$** |

*$p$ = 0.05/47 comparisons = 0.00106

**Table S10:** Spearman's correlation coefficients (rho) between *M. quahogii* (QPX) in clams or the environment and environmental metadata. Only correlations with $p < 0.05$, expressed as rho, *p*-value are shown. Positive correlations are shaded blue and negative are shaded red. Significant correlations are in bold using Bonferroni correction at $p < 0.001$ for 48 correlations for each parameter with metadata. For environmental parameters with more than one metric, only the strongest correlation is shown. *M. quahogii* abundance in SSW was not correlated with any parameter. Explanation of abbreviations for environmental parameters with multiple metrics (monthly means, sums or lags) can be found in Table S11.

| | Parameter | Day of Year | Month | Temperature | Salinity | DO | Chlorophyll | Precipitation | Wind Speed | Wind Direction |
|---|---|---|---|---|---|---|---|---|---|---|
| Clam Tissue (all) | %TPOS | NS | NS | LM3moT -0.376, 0.003 | NS | NS | NS | NS | NS | NS |
| | %POS | −0.378, 0.003 | −0.398, 0.0018 | **LM3moT −0.414, 0.001** | NS | NS | NS | NS | NS | NS |
| | %BLD | NS | NS | NS | NS | NS | NS | **−0.487, 0.00009** | NS | NS |
| | WP | −0.395, 0.002 | −0.398, 0.0017 | **LM3moT −0.428, 0.0007** | NS | NS | NS | NS | NS | NS |
| | Min | NS | NS | NS | NS | NS | NS | NS | Mean −0.275, 0.04 | NS |
| | Mean | −0.317, 0.01 | −0.358, 0.006 | M3moT −0.358, 0.005 | NS | BSW 0.359, 0.005 | BSW Chl b −0.275, 0.03 | NS | NS | NS |
| | Max | NS | −0.374, 0.003 | M3moT −0.384, 0.0026 | NS | NS | NS | NS | NS | NS |
| | Range | −0.391, 0.002 | **−0.416, 0.001** | **LM3moT −0.413, 0.001** | NS | NS | NS | NS | NS | NS |
| Clam Pallial Fluid (subset) | %TPOS | NS | NS | NS | SSW 0.554, 0.017 | NS | NS | NS | Mean −0.654, 0.003 | NS |
| | %POS | NS | NS | NS | NS | NS | NS | NS | NS | NS |
| | %BLD | NS | NS | NS | NS | NS | NS | NS | Mean −0.596, 0.009 | NS |
| | WP | NS | NS | NS | NS | NS | NS | NS | NS | NS |
| | Min | NS | NS | NS | ΔSSW-BSW 0.549, 0.018 | NS | NS | NS | NS | NS |
| | Mean | NS | NS | NS | | NS | NS | NS | NS | NS |
| | Max | NS | NS | NS | | NS | NS | NS | NS | NS |
| | Range | NS | NS | NS | BSW 0.47, 0.049 | NS | NS | M4moP −0.532, 0.023 | NS | NS |
| ENV | BSW | −0.29, 0.013 | −0.317, 0.007 | Max Air Temp 0.344, 0.003 | NS | ΔSSW-BSW 0.359, 0.002 | **BSW Chl a 0.444, 0.0001** | NS | Max 0.339, 0.0039 | 0.3, 0.01 |
| | SED | NS | NS | NS | **ΔSSW-BSW −0.383, 0.001** | NS | BSW Chl a −0.28, 0.019 | **S4moP 0.489, 0.00002** | NS | NS |

NS = not significant at $p < 0.05$

**Table S11:** Descriptions and abbreviations of data used in the correlation analyses.

| Full Description | Label | Quantitative (Q) or Category (C) |
|---|---|---|
| Clam QPX %Total Positive | TIS%Tpos | Q |
| Clam QPX %POS | TIS%Pos | Q |
| Clam %BLD | TIS%BLD | Q |
| Clam Minimum QPX copies/mg | TISMinQ | Q |
| Clam Maximum QPX copies/mg | TISMaxQ | Q |
| Clam Mean QPX copies/mg | TISAvgQ | Q |
| Clam Range QPX copies/mg | TISRangeQ | Q |
| Clam Weighted Prevalence | TISWPQ | Q |
| Site | Site | C |
| Embayment | Bay | C |
| Month | Month | C |
| QPX Disease History | QDisHis | C |
| Day of Year | Day | Q |
| Day of Both Years | DoBY | Q |
| Sediment QPX copies/mg | QPXsed | Q |
| BSW QPX copies/ml | QPXbsw | Q |
| SSW QPX copies/ml | QPXssw | Q |
| SSW Temperature (°C) | Tssw | Q |
| SSW Salinity (ppt) | Sssw | Q |
| SSW Dissolved Oxygen (mg/L) | DOssw | Q |
| BSW Temperature (°C) | Tbsw | Q |
| BSW Salinity (ppt) | Sbsw | Q |
| BSW Dissolved Oxygen (mg/L) | DObsw | Q |
| ΔTemp SSW-BSW | DelT | Q |
| ΔSalinity SSW-BSW | DelS | Q |
| ΔDO SSW – BSW | DelDO | Q |
| Depth (m) | Depth | Q |
| Depth | DepthC | C |
| SSW Total chl (ug/L) | TCssw | Q |
| SSW Chl a (ug/L) | CAssw | Q |
| SSW Chl b (ug/L) | CBssw | Q |
| SSW Chl c (ug/L) | CCssw | Q |
| BSW Total chl (ug/L) | TCbsw | Q |
| BSW Chl a (ug/L) | CAbsw | Q |
| BSW Chl b (ug/L) | CBbsw | Q |
| del Total Chl SSW-BSW | delTC | Q |
| Maximum Air Temperature | MaxAT | Q |
| Mean Air Temperature | MeanAT | Q |
| Min Air Temperature | MinAT | Q |
| Max Wind Speed (mph) | MaxWS | Q |
| Mean Wind Speed (mph) | MeanWS | Q |

**Table S11 (cont'd):** Descriptions and abbreviations of data used in the correlation analyses.

| Full Description | Label | Quantitative (Q) or Category (C) |
|---|---|---|
| Max Gust Speed (mph) | MaxGS | Q |
| Precipitation (in.) | Precip | Q |
| Cloud Cover | CloudC | Q |
| Weather Event | WEvent | C |
| Wind Direction (degrees) | WindDD | Q |
| Mean 1mo Temperature | M1moT | Q |
| Mean 2mo Temperature | M2moT | Q |
| Mean 3mo Temperature | M3moT | Q |
| Mean 4mo Temperature | M4moT | Q |
| Mean 1mo Precipitation | M1moP | Q |
| Mean 2mo Precipitation | M2moP | Q |
| Mean 3mo Precipitation | M3moP | Q |
| Mean 4mo Precipitation | M4moP | Q |
| Mean 1mo Wind Speed | M1moWS | Q |
| Mean 2mo Wind Speed | M2moWS | Q |
| Mean 3mo Wind Speed | M3moWS | Q |
| Mean 4mo Wind Speed | M4moWS | Q |
| Sum 1mo Precipitation | S1moP | Q |
| Sum 2mo Precipitation | S2moP | Q |
| Sum 3mo Precipitation | S3moP | Q |
| Sum 4mo Precipitation | S4moP | Q |
| Lag Mean 2mo Temperature | LM2moT | Q |
| Lag Mean 3mo Temperature | LM3moT | Q |
| Lag Mean 4mo Temperature | LM4moT | Q |

**Table S12:** Descriptive statistics of *M. quahogii* (QPX), total labyrinthulomycetes (LABY), and percent QPX of total labyrinthulomycetes in environmental samples. Values are expressed in terms of gene copies per mg sediment or ml seawater.

| **Parameter** | **QPX** | | | **LABY** | | | **%QPX** | | |
|---|---|---|---|---|---|---|---|---|---|
| Sample Type | **SED** | **BSW** | **SSW** | **SED** | **BSW** | **SSW** | **SED** | **BSW** | **SSW** |
| Mean | 93.15 | 241.44 | 7.36 | 17,666 | 24,612 | 16,578 | 0.71 | 0.85 | 0.08 |
| Standard Error | 16.06 | 78.81 | 2.92 | 1795 | 3584 | 3045 | 0.13 | 0.27 | 0.02 |
| Median | 22.1 | 9.84 | 0.54 | 11,667 | 15,389 | 9122 | 0.23 | 0.07 | 0 |
| Standard Deviation | 135.3 | 630.5 | 24.64 | 15,124 | 28,674 | 25,654 | 1.09 | 2.14 | 0.19 |
| Range | 702.61 | 2980 | 176.37 | 69,096 | 171,428 | 191,284 | 5.68 | 12.23 | 1.04 |
| Minimum | 0 | 0 | 0 | 487.48 | 720.94 | 780.83 | 0 | 0 | 0 |
| Minimum (non-zero) | 0.27 | 0.61 | 0.43 | 487.48 | 720.94 | 780.83 | 0.01 | 0 | 0 |
| Maximum | 702.61 | 2980 | 176.37 | 69,584 | 172,149 | 192,065 | 5.68 | 12.23 | 1.04 |
| Count | 71 | 64 | 71 | 71 | 64 | 71 | 71 | 64 | 71 |
| Confidence Interval (95.0%) | 32.03 | 157.49 | 5.83 | 3580 | 7162 | 6072 | 0.26 | 0.53 | 0.05 |

SED = sediment, BSW = bottom seawater, SSW = surface seawater
